# Assessing inconsistent responding in digital and paper formats: a replication study using the TIMSS 2023 Confidence in Mathematics scale in Ireland

**DOI:** 10.3389/fpsyg.2026.1817706

**Published:** 2026-04-23

**Authors:** Evi Konstantinidou, Vasiliki Pitsia, Aidan Clerkin, Michalis P. Michaelides

**Affiliations:** 1 Department of Psychology, University of Cyprus, Nicosia, Cyprus; 2 Educational Research Centre, Dublin, Ireland

**Keywords:** assessment mode, digital testing, ILSA, inconsistent responding, large-scale assessment, mixed-worded scales, mode effects, TIMSS

## Abstract

This study replicates and extends previous research on inconsistent responding in mixed-worded scales. Using a posttest-only experimental design, we examine the prevalence of inconsistent responding on the TIMSS 2023 Confidence in Mathematics Scale by comparing randomly equivalent digital and paper questionnaire administrations in Ireland. Two detection methods are employed: Mean Absolute Difference and Factor Mixture Analysis. Results indicate slightly higher rates of inconsistent responding in digital than in paper administration formats across most comparisons. Also, fourth-grade students exhibited higher rates of inconsistent responding compared to eighth-grade students. The study further investigates the role of student characteristics in predicting inconsistent responding. Among student background and digital-specific variables considered in binary logistic regression models, mathematics achievement was the only systematic negative predictor of inconsistent responding, explaining most of the variance, especially in younger students. This study highlights the importance of considering administration mode in large-scale assessments and provides implications for survey methodology, especially as digital testing becomes more widespread.

## 1 Introduction

Mixed-worded scales combine positively and negatively phrased items to measure the same underlying construct and are intended to prompt respondents to process item content more carefully (Podsakoff et al., 2003). However, there is limited empirical support for the effectiveness of using mixed-worded items, and research has shown that they often introduce substantial psychometric problems such as distorted factor structure and reduced measurement properties (Schmitt and Stuits, 1985; Steedle et al., 2019; Woods, 2006). Some of these problems may arise from inconsistent responding in mixed-worded scales, whereby individuals endorse both positively and negatively worded items in a similar manner even though they convey opposite meanings (Steinmann et al., 2022b). For example, in the Confidence in Mathematics scale used in this study, a student who reports high agreement with both “I usually do well in mathematics” and “Mathematics is harder for me than any other subject” would be considered to respond inconsistently, as the two items reflect opposing aspects of the same construct. Such response patterns are typically interpreted as reflecting insufficient cognitive processing, misunderstanding of item wording, rapid response or disengagement from the task.

To identify inconsistent respondents, two approaches have been mainly used in the literature. First, the Mean Absolute Difference (MAD) index compares the degree of agreement in responses to positive and negative items (Steedle et al., 2019). The index is calculated as the average absolute difference between responses to corresponding positively and negatively worded items (after reverse coding), with higher values indicating greater inconsistency. This approach provides a simple and transparent person-level indicator of inconsistency, but it requires the specification of a cut-off value to classify respondents into categories, which is inherently arbitrary.

An alternative approach, Factor Mixture Analysis (FMA), originates from factor analytic models (Lubke and Muthén, 2005) and uses latent class modeling to detect inconsistent response patterns (Steinmann et al., 2022b). FMA identifies latent subgroups of respondents based on their response patterns. This model-based approach allows for probabilistic classification into consistent and inconsistent classes and can capture more complex response behavior, although it is more computationally demanding and less straightforward to interpret.

A number of background characteristics have been linked to inconsistent responding. Lower achievement scores in reading or mathematics or lower cognitive ability have been associated with higher rates of inconsistency (e.g., Chen et al., 2024; Steinmann et al., 2022a). Younger students tend to respond more inconsistently than adolescents (e.g., Konstantinidou and Michaelides', 2025), and a recent study reported higher rates of inconsistent responding among boys, possibly due to lower average reading ability or lower conscientiousness (Steinmann et al., 2022a). Compared to students from higher socioeconomic backgrounds, disadvantaged students have shown higher levels of survey disengagement reflected in non-response behavior and lower internal consistency of scale responses in many countries participating in PISA 2018 (Buchholz, 2022).

The transition from paper-based to computer-based assessments has raised important questions about score comparability, fairness, and construct validity (Buerger et al., 2019), as differences in interface or digital familiarity may introduce construct-irrelevant variance (Lynch, 2022). Focusing on participants' response behavior in online and paper surveys, Magraw-Mickelson et al. (2022) found minimal differences in careless responding between modes, whereas Johnson (2005) reported lower-quality responses in online formats, including more missing data and longer strings of identical responses. Across equivalent samples, Konstantinidou and Michaelides' (2025) found that inconsistent responding on a paper-based questionnaire was slightly more pronounced among students who had completed the preceding assessment digitally rather than on paper, though results varied across the 16 countries examined and across grade levels.

The present study builds on the earlier work of Konstantinidou and Michaelides' (2025), where all students completed the questionnaire on paper regardless of the preceding achievement test mode. That study was based on a secondary analysis of data from the Bridge study of Trends in International Mathematics and Science Study (TIMSS) 2019, designed to evaluate potential mode effects arising from transitioning from paper to digital administration. One limitation, however, was that the questionnaire administration mode was not manipulated to match the achievement test mode. Ireland's administration of TIMSS 2023, which included a national Bridge study in addition to the main international data collection (McHugh et al., 2024), overcomes this limitation by implementing a fully aligned administration procedure. Specifically, students in Ireland who completed the TIMSS achievement test digitally also completed the student questionnaire digitally, while in the second condition, students completed both the test and the questionnaire on paper. This alignment ensures that any observed differences in inconsistent responding can be attributed solely to the mode of administration, rather than the confounding factor of transitioning between assessment modes, as was the case in the previous study that used TIMSS 2019 Bridge data.

The aim of this replication study was to determine whether the prevalence of inconsistent responding on the mixed-worded Confidence in Mathematics scale differs between the paper-based and digital administrations of TIMSS 2023 in Ireland. With a more refined experimental design, where examinees completed the entire TIMSS achievement test and questionnaire either digitally or on paper, the study offers stronger evidence on how administration mode influences response inconsistency in self-report mixed-worded instruments. Moreover, we extend prior work by examining which student demographic variables and process indicators (e.g., rapid guessing in the digital condition) predicted inconsistent responding. The study addresses the following research questions:

1. Does the prevalence of inconsistent responding on a mixed-worded scale differ between computer-based and paper-based questionnaire administrations among Grade 4 and Grade 8 students in Ireland?
2. Do MAD and FMA detection methods yield similar patterns of inconsistent responding across administration modes and grade levels?
3. Which student and process-related characteristics are associated with inconsistent responding on the Confidence in Mathematics scale, and do these associations differ across administration modes and grade levels?

## 2 Method

### 2.1 Sample

Approximately half of the countries participating in TIMSS transitioned from paper-based to digital testing in 2019. In contrast, Ireland moved to a digital administration in 2023. As part of the transition, Ireland administered a national Bridge study on paper to a separate sample of students alongside the main digital TIMSS assessment in order to monitor the possibility of mode effects (i.e., differences in student achievement that would be attributable to the different test administration format) arising during the transition.

The digital testing samples in Ireland comprised 4,735 Grade 4 students and 5,043 Grade 8 students, all of whom completed both an achievement test and a student questionnaire. The paper-based Bridge samples comprised 1,615 Grade 4 students and 1,231 Grade 8 students, who likewise completed both instruments (McHugh et al., 2024). Both samples were drawn from the same sampling frame using the same sampling procedures and can be considered *a priori* to be randomly equivalent (Slattery et al., 2025). Random assignment to the digital or paper-based administration condition took place at the school/classroom level. In total, at Grade 4, 137 schools administered in the digital mode only, 16 schools included a mixture of digital-testing and paper-testing classes, and 45 schools administered in the paper mode only. At Grade 8, 101 schools administered in the digital mode only, 52 schools included a mixture of digital-testing and paper-testing classes, and eight schools administered in the paper mode only (McHugh et al., 2024). All students in any given classroom completed the test and questionnaire under the same condition, either digitally or on paper.

Regardless of the mode of administration, the same questionnaire items were administered to all students. In line with the procedures developed during the international TIMSS 2019 Bridge study, the test booklets administered to the paper-based sample in TIMSS 2023 contained only the blocks of trend items that were common to the 2019 and 2023 cycles—approximately two-thirds of all test items—while the tests administered digitally contained these trend blocks as well as new items developed for digital administration. Importantly, students who completed the TIMSS achievement test in digital format also completed the student questionnaire digitally, whereas students who took the paper-based test completed the questionnaire on paper.

### 2.2 Measures

The Confidence in Mathematics scale was used for the identification of inconsistent respondents. It was presented to students as part of the student questionnaire and was delivered either digitally or on paper. The instrument consisted of seven items at Grade 4 (four phrased negatively) and eight items at Grade 8 (four phrased negatively). Sample items included statements such as “I usually do well in mathematics” (positive phrasing) or “Mathematics is harder for me than any other subject” (negative phrasing). Six of the items were presented identically to Grade 4 and Grade 8 students; one item was phrased differently across the two grades (“I am just not good at mathematics” at Grade 4 and “Mathematics is not one of my strengths” at Grade 8), and one additional item was presented to Grade 8 students (“I am good at explaining mathematics to others”). The wording of all items can be seen at https://timss2023.org/results/students-confident-math/. For all items, students indicated their level of agreement using a four-point response format ranging from “Agree a lot” to “Disagree a lot,” coded from 1 to 4.

To investigate the association between inconsistent responding and student characteristics, the following student-level variables were included in the analysis:

a) Gender (1 = girl; 2 = boy).
b) The Home Resources for Learning scale at Grade 4 and the Home Educational Resources scale at Grade 8, which are included as proxy indicators for students' socioeconomic status. The scales are constructed based on the number of books in a student's home; the highest education level of either parent; access to their own room and/or an internet connection at home; and (at Grade 4 only) the number of children's books at home and the highest occupation level of either parent. These are continuous measures (international mean = 10, international standard deviation = 2), with higher scores on the scales representing more resources for learning at home or higher socioeconomic status.
c) The language spoken at home (1 = always speak English or Irish at home; 2 = almost always, sometimes, or never speak English or Irish at home).
d) Students' digital self-efficacy (represented by a scale set to an international mean of 10 and standard deviation of 2, with higher scores representing higher perceived digital self-efficacy). This scale was constructed on the basis of students' responses to seven items (sample items: “I can tell if a website is trustworthy”; “I can write and edit text on a computer, tablet or smartphone”). Although this variable was available for both modes of administration, it was only used in the analysis for the digital administration mode.
e) An indicator of the extent to which students engaged in rapid guessing during the TIMSS mathematics test. This variable was available only in the digital administration mode, and was constructed as the count, for each student, of TIMSS mathematics test items that were answered in five or fewer seconds.
f) Mathematics achievement (represented by five plausible values that are scaled to an approximately normal distribution with an international centerpoint of 500 and a standard deviation of about 100 points).

### 2.3 Statistical analysis

The present study adopted a posttest-only experimental design, in which students are exposed to an achievement test in either paper or digital administration mode before completing the Confidence in Mathematics scale under the same mode. Following Konstantinidou and Michaelides' (2025), two methods were used to classify students as inconsistent or consistent respondents on the mixed-worded scale: the MAD index, applying the conventional threshold of 1.75, and an FMA model. All analyses incorporated TIMSS student sampling weights.

Differences in the prevalence of inconsistent responding were first examined across administration modes, comparing the proportions of inconsistent respondents between the paper-based and digital questionnaire conditions. We then explored grade-level differences within each mode by comparing Grade 4 and Grade 8 students. Finally, to investigate which factors were associated with inconsistent responding, we estimated binary logistic regression models (one model for each grade X mode condition). Predictors were entered hierarchically: (a) demographic characteristics, (b) variables related to digital behavior (only for the digital mode administration samples), and (c) mathematics achievement. Model fit was evaluated using Nagelkerke's *R*^2^. The outcome variable was coded as 0 for students classified as consistent responders and 1 for inconsistent responders.

The MAD was implemented in SPSS, while the FMA models were estimated in Mplus 8.11 (Muthén and Muthén, 1998–2017). A file with the MAD syntax and a sample file for the FMA code can be found on Open Science Framework. All binary logistic regression models were estimated in the IDB Analyzer (IEA, 2025), which automatically accounts for the TIMSS complex sampling design by applying the full student weights and the replicate weights. The software also processes the five plausible values for mathematics achievement simultaneously within a single estimation procedure.

## 3 Results

Table 1 summarizes the weighted prevalence of inconsistent responding in Ireland by grade level and questionnaire administration mode, based on both the MAD index and FMA. Using the MAD classification, the weighted proportion of inconsistent respondents in Grade 4 was 3.91% in the paper-based questionnaire group and 3.38% in the digital group, indicating broadly similar inconsistency rates across modes. Independent-samples proportions tests nevertheless showed that this difference was statistically significant (difference = −0.005, *SE* = 0.001, *Z* = −5.17, *p* < 0.001). Among Grade 8 students, the weighted prevalence was 2.18% in the paper mode and 2.41% in the digital mode, again showing only a minor difference between administration formats, although this difference was also statistically significant (difference = 0.002, *SE* = 0.001, *Z* = 2.87, *p* = 0.004).

**Table 1:** Number and percentage of inconsistent respondents in each grade level and administration mode.

Grade	Mode	Mean absolute difference			Factor mixture analysis	
		Unweighted		Weighted		
		*n* inconsistent	%	%	*n* inconsistent	Weighted %
4	Paper	51	3.19	3.91	99	6.19
	Digital	147	3.18	3.38	364	7.84
8	Paper	28	2.31	2.18	65	5.34
	Digital	141	2.85	2.41	331	6.67

Valid sample for MAD Grade 4 paper = 1,597; Grade 4 digital = 4,629; Grade 8 paper = 1,213; Grade 8 digital = 4,954.

The FMA approach yielded higher estimated rates of inconsistent responding across all subgroups compared to the MAD index. In Grade 4, 6.19% of paper respondents and 7.84% of digital respondents were classified as inconsistent, with the difference being statistically significant (difference = 0.017, *SE* = 0.001, *Z* = 11.84, *p* < 0.001). For Grade 8, the FMA-based estimates were 5.34% in the paper mode and 6.67% in the digital mode, again yielding a statistically significant difference (difference = 0.013, *SE* = 0.001, *Z* = 10.40, *p* < 0.001). Across both grades, FMA suggested slightly higher rates of inconsistency in the digital administration condition.

To examine which factors predict inconsistent responding, a series of binary logistic regression models were estimated separately by administration mode (paper-based vs. digital), grade level (Grade 4 vs. Grade 8), and inconsistency classification method (MAD vs. FMA). The results are presented in Table 2.

**Table 2:** Odds ratios from binary logistic regression models predicting inconsistent classification by grade level and administration mode.

Model predictors by administration mode	Grade 4						Grade 8					
	MAD			FMA			MAD			FMA		
	Step 1	Step 2	Step 3	Step 1	Step 2	Step 3	Step 1	Step 2	Step 3	Step 1	Step 2	Step 3
Paper												
Home resources	**0.79**	1.15		**0.77**	1.00		**0.65**	0.87		**0.70**	0.86	
Gender	0.74	0.69		0.79	0.73		1.06	0.84		1.04	0.90	
Language spoken at home	0.61	0.71	n/a	**0.55**	0.61	n/a	0.44	0.51	n/a	0.98	1.10	n/a
Mathematics achievement		**0.99**			**0.99**			**0.98**			**0.99**	
Nagelkerke *R*^2^	0.03	0.19		0.05	0.14		0.06	0.18		0.04	0.10	
Digital												
Home resources	**0.69**	**0.69**	0.98	**0.80**	**0.80**	1.04	**0.88**	0.88	1.12	**0.81**	**0.82**	1.00
Gender	0.70	0.75	0.69	0.95	0.96	0.88	0.96	0.99	0.82	1.07	1.09	0.92
Language spoken at home	0.97	0.96	1.01	0.91	0.91	0.98	1.46	1.45	1.37	1.19	1.22	1.16
Digital self-efficacy		1.05	**1.18**		0.97	1.04		1.04	1.07		1.02	1.04
Rapid guessing		**1.17**	1.08		1.04	0.98		1.06	1.02		1.05	1.02
Mathematics achievement			**0.99**			**0.99**			**0.99**			**0.99**
Nagelkerke *R^2^*	0.05	0.05	0.19	0.02	0.02	0.13	0.01	0.01	0.07	0.02	0.02	0.07

Statistically significant results (*p* < 0.05) shown in bold.

For the paper-based samples, the Grade 4 models explained a modest proportion of variance in inconsistent responding. Nagelkerke *R*^2^ values ranged from 0.03 to 0.19 for MAD-based inconsistency and from 0.05 to 0.14 for FMA-based inconsistency. Home resources emerged as a statistically significant predictor in both classification methods in Step 1, and language spoken at home was a statistically significant predictor in the FMA classification in Step 1, with higher home resources associated with lower odds of inconsistent responding (OR[MAD] = 0.79; OR[FMA] = 0.77) and students who always spoke English or Irish at home less likely to be classified as inconsistent respondents (OR = 0.55). However, neither remained statistically significant once mathematics achievement was introduced in Step 2. Mathematics achievement emerged as a consistent predictor, with higher achievement associated with lower odds of inconsistent responding (ORs ≈0.99). Girls and boys did not differ statistically significantly in their odds of being classified as inconsistent responders after other variables were taken into account in Steps 1 and 2 of the models.

Similarly, the Grade 8 models explained a modest proportion of variance in inconsistent responding. Nagelkerke *R*^2^ values ranged from 0.06 to 0.18 for MAD-based inconsistency and from 0.04 to 0.10 for FMA-based inconsistent responding. As in Grade 4, although home resources emerged as a statistically significant predictor in both classification methods in Step 1, with higher home resources associated with lower odds of inconsistent responding (OR[MAD] = 0.65; OR[FMA] = 0.70), it did not remain statistically significant once mathematics achievement was introduced in Step 2. Mathematics achievement was identified as a consistent predictor, with higher achievement associated with lower odds of inconsistent responding (OR[MAD] = 0.98; OR[FMA] = 0.99). Gender and language spoken at home accounted for only a small proportion of variance, indicating limited predictive value of sociodemographic variables at this grade level.

For the digital samples, the Grade 4 models explained a modest proportion of variance in inconsistent responding. For MAD-based inconsistency, Nagelkerke *R*^2^ increased from 0.05 in Step 1 to 0.19 in Step 3, while for FMA-based inconsistency *R*^2^ increased from 0.02 to 0.13. In the MAD-based model, several predictors were statistically significantly associated with inconsistent responding. Home resources were negatively related to inconsistent responding in Steps 1 and 2 (OR = 0.69), indicating lower odds of inconsistent responding among students with higher home resources. Rapid guessing also emerged as a statistically significant and positive predictor in Step 2 (OR = 1.17), but did not remain statistically significant in Step 3, while digital self-efficacy was statistically significantly positively associated with inconsistent responding in Step 3 (OR = 1.18). Mathematics achievement was statistically significantly and negatively related to inconsistent responding in Step 3 (OR = 0.99), indicating a higher likelihood of inconsistency among lower-achieving students. In contrast, in the FMA-based model at Grade 4, fewer variables were statistically significant predictors of inconsistent responding. Home resources remained statistically significantly and negatively associated with inconsistent responding in Steps 1 and 2 (OR = 0.80), while mathematics achievement was the only statistically significant predictor in the full model (OR = 0.99).

The Grade 8 models explained lower proportions of variance in inconsistent responding compared to the Grade 4 models. For both the MAD- and FMA-based models, Nagelkerke *R*^2^ values reached 0.07 in the full model. In the MAD-based models, home resources were statistically significantly and negatively associated with inconsistent responding in Step 1 (OR = 0.88), indicating lower odds of inconsistent responding among students with higher home resources. However, this relationship did not remain statistically significant in Steps 2 and 3. In the FMA-based models, home resources showed a similar pattern, being statistically significantly associated with inconsistent responding in Steps 1 and 2 but not reaching statistical significance in Step 3 when mathematics achievement was introduced.

## 4 Discussion

This study replicates and extends Konstantinidou and Michaelides' (2025) analysis of inconsistent responding in TIMSS 2019 data. Here, by overcoming a limitation of that study through the use of large student samples from Ireland who participated in TIMSS 2023 in fully digital or fully paper-based administrations, inconsistent responding was found to be generally low in both grade levels.

In three out of four grade-by-detection method comparisons, inconsistent responding was slightly more common in computer-based than in paper-based formats. These differences were statistically significant, but their magnitude was small. As expected, inconsistency rates were higher among younger students, with recent evidence suggesting that older students are less likely than younger students to respond inconsistently to mixed-worded scales, possibly because more developed reading and cognitive skills help them handle changing item wording more effectively (Steinmann et al., 2024). This developmental pattern is consistent with the original study by Konstantinidou and Michaelides' (2025). Using the MAD method, inconsistent responding was relatively low, but the FMA approach revealed higher rates of inconsistency, particularly in Grade 4 samples. This finding also replicates the original findings, reinforcing the idea that the method of detection is crucial for understanding response patterns.

Mathematics achievement was a key predictor of inconsistent responding. In both grade levels, lower-achieving students were more likely to respond inconsistently in the questionnaire, as evidenced by the statistically significant negative association between mathematics achievement and inconsistent responding. Considering this achievement variable as a proxy for cognitive ability, the finding is in agreement with studies correlating cognitive abilities with consistent and thoughtful responding (Michaelides et al., 2026; Steedle et al., 2019; Steinmann et al., 2022b). In mixed-worded scales, negative items seem to require more cognitive resources for comprehending an item (Koutsogiorgi and Michaelides, 2022).

The inclusion of digital-specific variables, such as digital self-efficacy and rapid guessing, in the models for the digital samples showed some statistically significant positive associations with inconsistent responding in Grade 4 only when mathematics achievement was not included in the model. One exception to this pattern was found in the MAD approach, where digital self-efficacy remained a statistically significant positive predictor of inconsistent responding even after mathematics achievement was accounted for. However, in Grade 8, the digital-specific variables were not statistically significantly associated with inconsistent responding. In both grade levels, student gender, home resources, and language spoken at home were not statistically significant predictors when mathematics achievement was included in the models. This suggests that achievement may act as the primary predictor of inconsistent responding, overshadowing the role of background characteristics.

This replication study demonstrates that questionnaire administration mode has a small but consistent influence on inconsistent responding in mixed-worded scales, with digital formats generally yielding slightly higher rates than paper formats. The fully aligned design, where students completed both the achievement test and questionnaire under the same mode, strengthens these conclusions by removing the confounding effect of mode switching present in the original work by Konstantinidou and Michaelides' (2025). Although the differences are modest, the pattern is robust across grade levels and detection methods, underscoring the importance of considering administration mode when evaluating data quality in self-report mixed-worded scales, particularly as large-scale assessment programs continue to transition to digital delivery. Future studies could explore whether motivation, personality, or prior digital experience moderates the effect of mode on inconsistent responding, as well as designing interventions to encourage thoughtful and engaged responding, even with younger students.

## Data Availability

The data analyzed in this study is subject to the following licenses/restrictions: Data from the national bridge study (paper-based sample) are available upon request. Requests to access these datasets should be directed to Aidan Clerkin, aidan.clerkin@erc.ie or info@erc.ie. Data for the digital TIMSS 2023 sample in Ireland are available from https://www.iea.nl/data-tools/repository/timss.
